# FADEL: Ensemble Learning Enhanced by Feature Augmentation and Discretization

**DOI:** 10.3390/bioengineering12080827

**Published:** 2025-07-30

**Authors:** Chuan-Sheng Hung, Chun-Hung Richard Lin, Shi-Huang Chen, You-Cheng Zheng, Cheng-Han Yu, Cheng-Wei Hung, Ting-Hsin Huang, Jui-Hsiu Tsai

**Affiliations:** 1 Department of Computer Science and Engineering, National Sun Yat-sen University, Kaohsiung 804, Taiwan; cshung@g-mail.nsysu.edu.tw (C.-S.H.); yczheng7652@gmail.com (Y.-C.Z.); yu199483@gmail.com (C.-H.Y.); nick47052@gmail.com (C.-W.H.); guys0510@gmail.com (T.-H.H.); 2 Artificial Intelligence Research and Promotion Center, National Sun Yat-sen University, Kaohsiung 804, Taiwan; 3 Department of Computer Science and Information Engineering, Shu-Te University, Kaohsiung 824, Taiwan; shchen@stu.edu.tw; 4 Division of Cardiology, Department of Internal Medicine, Kaohsiung Chang Gung Memorial Hospital, Kaohsiung 833, Taiwan; 5 School of Medicine, Tzu Chi University, Hualien 970, Taiwan; 6 Department of Psychiatry, Dalin Tzu Chi Hospital, Buddhist Tzu Chi Medical Foundation, Chia-Yi 622, Taiwan

**Keywords:** imbalance class classification, data augmentation, feature augmentation, feature discretization, ensemble learning

## Abstract

In recent years, data augmentation techniques have become the predominant approach for addressing highly imbalanced classification problems in machine learning. Algorithms such as the Synthetic Minority Over-sampling Technique (SMOTE) and Conditional Tabular Generative Adversarial Network (CTGAN) have proven effective in synthesizing minority class samples. However, these methods often introduce distributional bias and noise, potentially leading to model overfitting, reduced predictive performance, increased computational costs, and elevated cybersecurity risks. To overcome these limitations, we propose a novel architecture, FADEL, which integrates feature-type awareness with a supervised discretization strategy. FADEL introduces a unique feature augmentation ensemble framework that preserves the original data distribution by concurrently processing continuous and discretized features. It dynamically routes these feature sets to their most compatible base models, thereby improving minority class recognition without the need for data-level balancing or augmentation techniques. Experimental results demonstrate that FADEL, solely leveraging feature augmentation without any data augmentation, achieves a recall of 90.8% and a G-mean of 94.5% on the internal test set from Kaohsiung Chang Gung Memorial Hospital in Taiwan. On the external validation set from Kaohsiung Medical University Chung-Ho Memorial Hospital, it maintains a recall of 91.9% and a G-mean of 86.7%. These results outperform conventional ensemble methods trained on CTGAN-balanced datasets, confirming the superior stability, computational efficiency, and cross-institutional generalizability of the FADEL architecture. Altogether, FADEL uses feature augmentation to offer a robust and practical solution to extreme class imbalance, outperforming mainstream data augmentation-based approaches.

## 1. Introduction

In the fields of machine learning and data mining, one of the most persistent challenges in binary classification tasks is the issue of imbalanced data [1]. When the number of positive instances is significantly lower than that of negative instances, conventional machine learning algorithms often become biased toward the majority class, which results in a failure to effectively identify or predict critical patterns associated with the minority class [2,3]. This imbalance is prevalent across a wide range of real-world applications, including rare disease diagnosis in healthcare [4,5], fraud detection in finance [6], and fault prediction in industrial systems [7]. Such skewed distributions not only undermine the model’s generalizability and stability across datasets but also create a misleading impression of high overall accuracy. In extreme cases, the model may perform exceedingly poorly in detecting minority class instances [8,9], sometimes failing to identify any positive cases at all—a phenomenon commonly referred to as model collapse. This poses substantial risks in practice, particularly in scenarios where accurate detection of rare but critical events is essential. Consequently, improving a model’s ability to recognize minority class instances under imbalanced distributions has become a central focus in contemporary machine learning research.

To address this issue, researchers have traditionally employed a variety of data-level balancing techniques. Among the most widely adopted are oversampling, undersampling, and their various derivatives. Notable examples include the Synthetic Minority Over-sampling Technique (SMOTE) [10] and Adaptive Synthetic Sampling (ADASYN) [11]. These methods synthesize new minority class samples by interpolating between existing data points or applying distance-based metrics. The primary aim is to expand the decision boundaries of the minority class and, in doing so, enhance the model’s capacity to identify underrepresented patterns.

While these techniques can improve recall to some extent, they often introduce limitations such as noisy sample generation, class overlap, or model overfitting—particularly in high-dimensional or sparsely distributed datasets. These challenges are further amplified when the data manifold is complex or poorly structured. In response, recent studies have explored the use of Generative Adversarial Networks (GANs) [12] for synthetic data generation in tabular domains. Prominent among these are the Conditional Tabular GAN (CTGAN) [13] and Tabular Variational Autoencoder (T-VAE), with the latter demonstrating superior performance in preserving the statistical integrity of both continuous and categorical features during data reconstruction. However, both approaches require additional model training, non-trivial hyperparameter tuning, and considerable computational overhead, which can limit their practicality in resource-constrained environments.

In parallel with data-level balancing efforts, a growing body of research has shifted toward algorithm-level strategies and ensemble learning frameworks aimed at improving minority class prediction without modifying the underlying data distribution [14,15,16]. Among these, stacking has emerged as a particularly effective approach. This technique combines the predictions of multiple base learners through a second-layer meta-model, theoretically allowing the ensemble to leverage the unique strengths of each individual model. Prior research has shown that stacking frameworks can yield enhanced recall and specificity compared to single-model classifiers, particularly in imbalanced learning contexts [16,17,18].

However, existing stacking architectures typically apply a globally uniform feature preprocessing strategy—such as standardization or full discretization—across all base models. While effective for homogeneous datasets consisting of purely continuous or ordinal variables [19,20], this approach becomes problematic in cases involving heterogeneous feature types, such as skewed continuous features alongside categorical or ordinal variables. Uniform preprocessing can result in information loss and reduce model sensitivity to intrinsic feature distributions. For instance, discretizing all continuous variables may benefit tree-based models by enhancing interpretability, but it can impair the performance of algorithms like XGBoost [21] and LightGBM [22], which are optimized to exploit numerical precision. Conversely, retaining continuous features in raw form may hinder the performance of models such as CatBoost [23], which are designed to operate effectively on categorical and ordinal data.

Additionally, many stacking-based systems assume that the training data have already been balanced using SMOTE, ADASYN, or GAN-derived synthetic samples. This reliance on prior data augmentation limits the applicability of such methods in scenarios where data balancing is infeasible or undesirable due to cost, security, or deployment constraints. In other words, conventional ensemble learning frameworks often fail to directly address the fundamental challenge of improving minority class recognition without synthetic data.

Building upon these challenges and their associated limitations, the primary motivation of this study is to develop a stacking-based learning framework that eliminates the need for data-level balancing or data augmentation techniques, while simultaneously leveraging the distinct strengths of both continuous and ordinal features to enhance minority class prediction. Existing methods often depend on synthetic sample generation and uniform feature preprocessing pipelines, which increase complexity and computational cost, and may distort the original data distribution—ultimately degrading data quality. To overcome these limitations, this study proposes a novel feature augmentation framework called FADEL. The FADEL approach embeds feature-type-aware processing strategies directly within the model architecture to overcome the structural limitations of conventional stacking.

The FADEL architecture comprises two key components. The first layer consists of four base models—XGBoost, LightGBM, CatBoost, and AdaBoost [24]—with feature inputs dynamically routed based on each model’s compatibility with different feature types. For gradient boosting algorithms such as XGBoost and LightGBM, raw continuous features are preserved to exploit their capability in capturing fine-grained numerical relationships and complex non-linear boundaries. In contrast, for CatBoost and AdaBoost, continuous features are first discretized via a supervised method, transforming them into interval-based representations that support effective modeling of categorical thresholds and latent distribution patterns. This design avoids the pitfalls of global preprocessing pipelines and allows each classifier to operate under conditions most suited to its strengths.

The second layer of FADEL employs a Random Forest as the meta-model to integrate predictions from the base learners. This design balances recall for the minority class and precision for the majority class. Unlike traditional approaches that rely on preprocessing steps such as SMOTE or GAN-based augmentation, FADEL improves recall, F1-score, and G-mean under severely imbalanced settings solely through feature-type-aware routing and algorithm-level integration. Furthermore, the model demonstrates strong performance on external validation datasets, highlighting its generalizability and potential for real-world deployment.

In summary, FADEL simplifies the preprocessing and resampling procedures commonly required for handling imbalanced data, reducing both computational burden and model complexity. Theoretically, it addresses the key limitations of traditional stacking frameworks, which often struggle to handle feature heterogeneity and minority class detection simultaneously. Unlike existing approaches that depend on synthetic data generation (e.g., SMOTE, ADASYN, CTGAN), FADEL operates directly on the raw data, thereby avoiding data distortion, reducing risks of overfitting and data poisoning, and preserving the integrity of the original dataset. The result is a robust and adaptable solution for extreme imbalanced classification problems, offering both practical and theoretical advancements in ensemble learning.

The remainder of this paper is organized as follows. Section 2 reviews the relevant literature on imbalanced data learning, feature discretization methods, and ensemble modeling strategies. Section 3 presents the proposed feature discretization workflow and the architectural design of the FADEL model. Section 4 outlines the experimental setup, evaluation metrics, and comparative results. Section 5 discusses the implications of the findings. Section 6 addresses the limitations of this study and proposes directions for future research. Finally, Section 7 concludes the paper and summarizes its key contributions.

## 2. Related Works

This study proposes a novel FADEL framework that effectively improves minority class recognition without relying on any data-level balancing preprocessing. Accordingly, Section 2 is dedicated to reviewing three core research areas that form the foundation of this work: (1) Techniques for Handling Class Imbalance in Machine Learning, (2) Feature Discretization and Optimal Interval Selection Methods, and (3) Ensemble Learning and Multi-Model Integration Strategies.

### 2.1. Techniques for Handling Class Imbalance in Machine Learning

Imbalanced datasets have long posed significant challenges to the classification performance of machine learning models. Early approaches primarily relied on Random Oversampling (ROS) and Random Undersampling (RUS) [25]. While these techniques helped balance class distributions, their inherent randomness often led to overfitting or the loss of critical majority-class information. To overcome these limitations, Chawla et al. [10] introduced the Synthetic Minority Over-sampling Technique (SMOTE), which creates synthetic minority samples by interpolating between existing instances, thereby enhancing the recognition of class boundaries [26]. However, subsequent studies have reported that SMOTE can introduce noise and cause class overlap, particularly in datasets with complex or non-linear distributions [27].

To address these issues, He et al. [11] proposed Adaptive Synthetic Sampling (ADASYN), which adjusts the oversampling rate based on the learning difficulty of individual minority class samples. This method shifts the decision boundary toward harder-to-learn regions, effectively reducing the bias introduced by class imbalance. Other SMOTE-based extensions, such as SMOTE-ENC [28], have been designed to support datasets with mixed feature types, including both categorical and continuous variables. Meanwhile, Zhu et al. [29] introduced the OREM method, which incorporates local density information to improve the quality of synthetic samples and enhance model generalization. Nevertheless, a major drawback of these data-level approaches lies in their heavy reliance on generating large numbers of synthetic samples, which substantially increases computational cost and complicates hyperparameter optimization [14,15].

In response to these challenges, researchers have explored the application of Generative Adversarial Networks (GANs) for handling imbalanced data. CTGAN [13], for example, produces realistic tabular samples that preserve the statistical characteristics of the original data, while TVAE [13] focuses on improving reconstruction fidelity, especially for continuous features. More recently, CTGAN-MOS [30] has been proposed to incorporate noise filtering and adaptive fusion mechanisms, effectively reducing the number of synthetic samples while enhancing G-mean performance. However, its complex six-stage pipeline—from data preprocessing to noise suppression—introduces considerable system overhead, limiting its feasibility in practical, real-world deployments [30].

In parallel, undersampling techniques have also gained traction as a means to mitigate class imbalance. For instance, Yan et al. proposed Spatial Distribution-based Undersampling (SDUS) [31], which preserves the structural integrity of the majority class by constructing spherical neighborhoods and applying a two-stage instance selection strategy. Similarly, Wang et al. developed Entropy and Confidence-Based Undersampling Boosting (ECUBoost) [32], which dynamically filters majority class samples using entropy metrics and confidence scores, thereby improving model sensitivity and generalization. These methods—including neural network-based undersampling [33], spatial-guided selection [31], and entropy-confidence hybrid filtering [32]—have significantly contributed to the theoretical foundations of majority class sample selection.

Collectively, the aforementioned techniques have demonstrated substantial improvements in sensitivity, AUC, and F1-score across a wide range of imbalanced learning scenarios [25,26,27,28,29,30,31,32,33]. However, they remain heavily dependent on either synthetic data generation or computationally intensive instance selection strategies—both of which increase resource requirements and system complexity. As a result, there is a growing need for alternative solutions that can address class imbalance without relying on data augmentation or uniform preprocessing. Therefore, this study aims to transcend the limitations of traditional frameworks by proposing a model-level integration strategy that operates directly on raw input features, accommodates feature heterogeneity, and enhances minority class recognition under highly imbalanced conditions—without introducing the burdens of synthetic sampling or complex instance filtering.

### 2.2. Feature Discretization and Optimal Interval Selection Methods

Feature discretization and optimal interval selection pose unique challenges in the context of imbalanced datasets. Early work by Liu et al. [34] demonstrated that discretization can transform continuous attributes into a finite set of intervals, thereby enhancing model interpretability and reducing computational complexity. However, Rajbahadur et al. [35] identified a critical limitation: when discretization is based on manually defined thresholds, it may introduce discretization noise. This issue is particularly problematic for data points near interval boundaries, as they are more prone to misclassification—especially within the minority class. In a comprehensive review, Garcia and Luengo [36] categorized a wide range of discretization algorithms and emphasized the effectiveness of supervised criteria—such as information entropy, chi-squared statistics, and probabilistic metrics—for improving classification performance. Nevertheless, these metrics are inherently sensitive to class distribution. In highly imbalanced scenarios, the dominant majority class can bias the selection of cut points, resulting in the merging or exclusion of narrow intervals that may contain crucial information about the minority class. This effect is considered one of the key reasons why traditional discretization approaches often fail to deliver robust predictive performance under extreme imbalance.

To address these limitations, a class-dependent discretization method was proposed, which automatically determines the number of intervals by maximizing the statistical dependency between intervals and class labels. While this strategy partially mitigates information loss by aligning discretization with class-specific patterns, its effectiveness still depends on the availability of a sufficient number of minority class samples. In highly imbalanced datasets, the scarcity of minority instances often leads to unstable dependency estimates, thereby compromising the reliability of cut-point selection. Similarly, Thaiphan and Phetkaew [37] introduced a comparative framework that categorizes datasets based on class imbalance ratios—distinguishing between similar and dissimilar class proportions. Their evaluation of algorithms such as the Minimum Description Length Principle (MDLP) and the Class-Attribute Contingency Coefficient (CACC) within decision tree classification contexts provides valuable insights into performance variations across different data distributions. However, their analysis primarily emphasizes overall classification performance rather than offering targeted assessments of minority class behavior under severe imbalance.

Xie and Wu [38] advanced the field by proposing Multi-Scale Interval-Set Decision Tables, which utilize forward complementary conditional entropy to identify optimal intervals via multi-scale analysis. Although the approach offers strong theoretical rigor, its applicability to high-dimensional, imbalanced data remains limited due to computational overhead. In a parallel effort, Liu and Wang [39] introduced a heterogeneity-based criterion that seeks to improve the recognition of class mixtures within intervals, offering an alternative to traditional entropy-based methods. Additionally, studies by Alazaidah [40], and Blessie and Karthikeyan [41] demonstrated that combining supervised discretization with auxiliary strategies—such as feature selection or hybrid filtering—can further improve classification accuracy.

Although primarily developed for oversampling, Zhang et al.’s FSDR-SMOTE [42] provides useful insights relevant to discretization. Their method leverages feature standard deviation to identify boundary samples, effectively using distributional spread as a proxy for instance importance. This concept aligns closely with the goal of identifying optimal discretization cut points, revealing an implicit connection between distribution-aware sampling strategies and effective discretization. Collectively, these findings underscore the underexplored potential of using feature distribution metrics to improve class boundary recognition under imbalance.

In summary, while existing discretization methods have achieved notable progress in information retention and overall classification accuracy, few studies have systematically examined whether discretization and interval optimization—without any form of data augmentation—can independently enhance minority class prediction in highly imbalanced scenarios. This study directly addresses this gap by proposing an integrated approach that combines supervised discretization with class-dependent interval selection. The goal is to improve minority class recognition through a distribution-aware, model-level solution that avoids reliance on synthetic sampling or extensive data preprocessing.

### 2.3. Ensemble Learning and Multi-Model Integration Strategies

Galar et al. [2] reviewed the application of Bagging, Boosting, and hybrid ensemble methods in the context of imbalanced classification tasks. Their analysis emphasized that while combining random undersampling with boosting techniques—such as SMOTEBoost and RUSBoost—can enhance the recognition of minority class instances, these methods are heavily reliant on data-level resampling. Such dependence often distorts the original data distribution, potentially compromising model generalizability. Building on this, Sagi and Rokach [43] highlighted the unique potential of Stacking, which integrates multiple diverse base learners and benefits from error compensation across models. However, they also noted that most stacking implementations fail to simultaneously account for feature heterogeneity without resorting to synthetic data generation or class balancing procedures.

Complementing this line of inquiry, Mienye and Sun [44] provided a comprehensive review of ensemble learning strategies and acknowledged the effectiveness of advanced gradient boosting algorithms such as XGBoost, LightGBM, and CatBoost across a broad range of applications. Nevertheless, their analysis also identified a critical limitation: existing frameworks generally lack adaptive mechanisms for accommodating diverse feature types, a shortcoming that becomes particularly problematic when dealing with heterogeneous tabular data containing both continuous and categorical variables.

In parallel, exploratory undersampling techniques such as EasyEnsemble and BalanceCascade have been proposed to mitigate majority class bias by training multiple classifiers on disjoint subsets of the majority class. Although these techniques improve AUC and F-measure, their scalability is hindered by significant data reduction and the computational cost of repeated model training. Further examining the limitations of resampling, Altalhan et al. [45] pointed out that in high-dimensional or complex feature spaces, both oversampling and undersampling approaches often introduce additional noise, leading to model instability. To address these concerns, Zhu et al. [46] proposed imDEF, a dynamic ensemble learning framework that integrates synthetic data generation and stepwise reinforcement learning to improve minority class recall. However, despite its innovative architecture, imDEF still fundamentally depends on synthetic sample generation to diversify base learners, thereby increasing overall system complexity and tuning requirements.

In response to the limitations of both data-level resampling and conventional stacking frameworks, this study proposes a novel architecture—FADEL. The FADEL framework is explicitly designed to exploit feature-type diversity without relying on data augmentation or resampling. Specifically, raw continuous features are preserved for input into XGBoost and LightGBM, allowing these models to fully leverage their strengths in capturing complex, non-linear decision boundaries. Simultaneously, the same continuous features are discretized using a supervised strategy prior to being input into CatBoost and AdaBoost, which are more effective with categorical or interval-scaled features. A Random Forest meta-model is then employed to integrate the predictions of all base learners.

By strategically routing different feature representations to classifiers most compatible with their structure, FADEL embraces both feature heterogeneity and model diversity while completely avoiding synthetic sample generation or instance resampling. Experimental results confirm that FADEL consistently achieves high sensitivity and robust predictive performance across various imbalance settings, outperforming traditional ensemble models that rely heavily on data-level balancing techniques. This demonstrates that model-level integration and feature-type awareness can jointly offer a powerful alternative to conventional resampling-based solutions in imbalanced learning.

## 3. Methods

**Figure 1:**
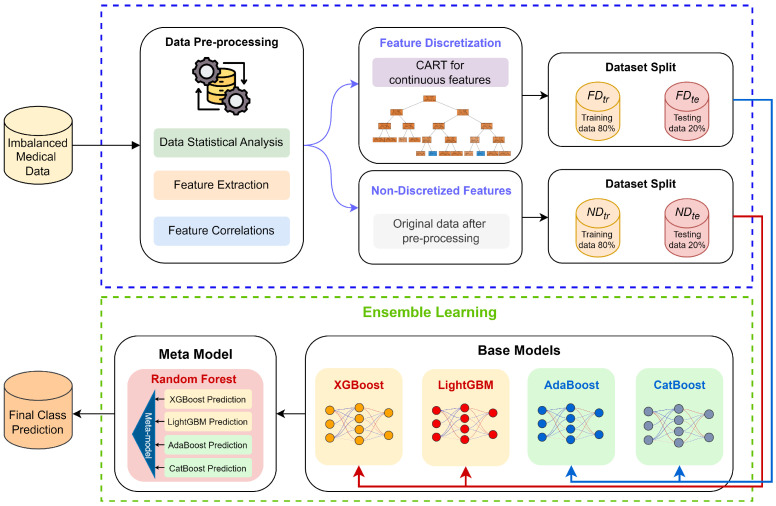
The overall workflow of the FADEL framework.

An overview of the proposed FADEL architecture is first presented. The methodology developed in this study comprises three key stages: feature discretization, base models construction, and ensemble learning-based prediction. The subsequent sections detail the design rationale and procedural workflow of each component. For ease of interpretation, the primary notations and their corresponding mathematical definitions are summarized in Table 1, while Figure 1 illustrates the overall architecture of the FADEL framework.

### 3.1. Framework Overview

The proposed FADEL framework is specifically designed to address the challenges posed by highly imbalanced datasets. Unlike conventional methods that rely on data-level balancing techniques—such as oversampling, undersampling, or generative data augmentation—FADEL overcomes these limitations through a two-stage architecture that integrates heterogeneous feature processing with ensemble learning to enhance minority class recognition.

In the first stage, the framework dynamically adjusts the treatment of continuous features based on each base model’s sensitivity to feature types. For gradient boosting algorithms such as XGBoost and LightGBM, which excel at capturing complex non-linear decision boundaries, the original continuous features are retained to preserve fine-grained distributional characteristics. Conversely, for models like CatBoost and AdaBoost—more adept at handling categorical, ordinal, or discretized features—a supervised, decision tree-based discretization method is applied to transform continuous variables into interval-based representations. This transformation not only improves interpretability but also reveals latent risk patterns embedded within the feature space.

Once each base model has been individually trained, it produces class probability estimates for every instance. These outputs are concatenated into a probability vector ensemble, which is then passed to the second stage. At this stage, a Random Forest meta-model is employed, leveraging both random subspace sampling and bootstrap aggregation. This approach enhances model robustness and generalization in predicting the minority class, while mitigating individual biases of the base learners.

Crucially, unlike traditional stacking methods that often require balanced class distributions or uniform feature preprocessing, FADEL enables direct learning from raw, imbalanced data. By preserving feature-type diversity and avoiding synthetic augmentation, the framework harnesses the unique strengths of each base learner within the ensemble.

The proposed architecture has demonstrated high sensitivity, specificity, and robustness in real-world clinical applications characterized by severe class imbalance, underscoring its practical utility in critical domains such as rare disease prediction. Figure 1 illustrates the overall structure of the FADEL framework.

### 3.2. Feature Discretization Based on CART Decision Tree

One of the essential components underpinning the FADEL framework is transforming continuous features through discretization. This section details the discretization process based on the CART decision tree. Specifically, it transforms continuous variables into a finite set of interval-based or categorical representations. Beyond simplifying data structure, discretization enhances feature interpretability, which is particularly critical for improving prediction accuracy in highly imbalanced datasets—without relying on data-level balancing or synthetic augmentation. This transformation highlights the relative density of minority class samples within certain intervals, thereby enabling models to better detect patterns associated with rare-class characteristics.

Moreover, in datasets exhibiting high skewness or uneven distributions, discretization helps reduce the impact of outliers and noise, thereby mitigating the adverse effects of skewed distributions on model performance. Owing to these benefits, discretization has increasingly been recognized as an effective strategy for handling class imbalance and enhancing model generalizability in recent machine learning research. In this study, we compare unsupervised discretization methods—such as equal-width binning and equal-frequency binning—with a supervised discretization approach based on decision tree partitioning, applying each method independently to every continuous feature to evaluate their effectiveness.

The discretization method proposed in this study adopts a supervised CART (Classification and Regression Tree) approach, which partitions the feature space by identifying threshold values at the internal nodes of the tree. To regulate the granularity of the resulting intervals, the maximum tree depth and maximum number of leaf nodes are used as control parameters. This discretization not only prevents overfitting caused by excessively deep trees, but also ensures that the resulting partitions are stable and semantically meaningful.

For a given continuous feature *j*, we define the set of observed values as $X_{j}={\left\{x_{j}^{\left(i\right)}\right\}}_{i=1}^{N}$ where $x_{j}^{\left(i\right)}$ denotes the value of the *j*-th feature for the *i*-th sample. To incorporate class label information, we construct a feature-label paired dataset and train a CART model $T_{j}$ as follows $T_{j}=\mathrm{CART}\left({\left\{\left(x_{j}^{\left(i\right)},\;y_{i}\right)\right\}}_{i=1}^{N}\right)$.

Unlike unsupervised discretization methods that rely solely on the distribution of feature values, the supervised approach considers the distribution of class labels, thereby producing intervals that are more informative for classification tasks.

To avoid over-partitioning that could hinder generalization, we constrain the tree by predefining its maximum depth $D_{j}$ and maximum number of leaf nodes $L_{j}$. These hyperparameters can be selected based on the number of bins used in equal-width or equal-frequency discretization methods, or tuned via cross-validation to strike a balance between classification performance and model stability. The final number of discretized intervals, $K_{j}$, is therefore constrained as:

(1) $$\begin{array}{c} K_{j}\leq \min\left\{2^{D_{j}},\;L_{j}\right\} \end{array}$$

After training the CART decision tree $T_{j}$, we extract all valid splitting thresholds from the tree and sort them in ascending order to form the threshold vector:

(2) $$\begin{array}{c} \theta_{j}=\left[\theta_{j}^{\left(1\right)}<\theta_{j}^{\left(2\right)}<\cdots <\theta_{j}^{\left(K_{j}-1\right)}\right] \end{array}$$

where $\theta_{j}^{\left(k\right)}$ denotes the *k*-th splitting threshold that defines the boundaries between adjacent intervals. To formally define the range of each interval, we further extend the boundaries as:

(3) $$\begin{array}{c} \theta_{j}^{\left(0\right)}=-\infty ,\;\theta_{j}^{\left(K_{j}\right)}=+\infty \end{array}$$

Based on the ordered set of thresholds, the original feature domain can be partitioned into $K_{j}$ disjoint intervals. Each interval is defined as:

(4) $$\begin{array}{c} I_{j}^{\left(k\right)}=\left\{x\in R\;|\;\theta_{j}^{\left(k-1\right)}<x\leq \theta_{j}^{\left(k\right)}\right\},\;k=1,\ldots ,K_{j} \end{array}$$

where $I_{j}^{\left(k\right)}$ represents the *k*-th discretized interval.

For computation purposes, we define an indicator function $I[x\in I_{j}^{\left(k\right)}]$ to determine whether a given sample value *x* falls within the *k*-th interval. This indicator function can be decomposed as:

(5) $$\begin{array}{c} \begin{array}{cc} I[x\in I_{j}^{\left(k\right)}] & =I[\theta_{j}^{\left(k-1\right)}<x\leq \theta_{j}^{\left(k\right)}]\\ & =I\left[x>\theta_{j}^{\left(k-1\right)}\right]\cdot I\left[x\leq \theta_{j}^{\left(k\right)}\right]\\ & =\left[1-I[x\leq \theta_{j}^{\left(k-1\right)}]\right]\cdot I\left[x\leq \theta_{j}^{\left(k\right)}\right]\\ & =I\left[x\leq \theta_{j}^{\left(k\right)}\right]-I\left[x\leq \theta_{j}^{\left(k-1\right)}\right] \end{array} \end{array}$$

where I[·] denotes the indicator function, which returns 1 if the condition inside is true, and 0 otherwise. To map a continuous value to its corresponding interval index, we define the discretization mapping function as follows:

(6) $$\begin{array}{c} C_{j}:R\to \left\{1,2,\ldots ,K_{j}\right\} \end{array}$$

with the mapping rule defined as:

(7) $$\begin{array}{c} C_{j}\left(x\right)=k\;\mathrm{if}\;\mathrm{and}\;\mathrm{only}\;\mathrm{if}\;x\in I_{j}^{\left(k\right)} \end{array}$$

where $C_{j}\left(\cdot \right)$ maps the input value *x* to the corresponding interval index.

For computational efficiency, the mapping can also be expressed using a summation form:

(8) $$\begin{array}{c} C_{j}\left(x\right)=\sum_{k=1}^{K_{j}}k\cdot I\left[x\in I_{j}^{\left(k\right)}\right] \end{array}$$

For each sample $x_{j}^{\left(i\right)}$, the discretized result is denoted as:

(9) $$\begin{array}{c} z_{j}^{\left(i\right)}=C_{j}\left(x_{j}^{\left(i\right)}\right) \end{array}$$

where $z_{j}^{\left(i\right)}$ represents the interval index for the *i*-th sample on the *j*-th feature.

If there are *J* continuous features to be discretized, the final discretized feature vector for the *i*-th sample is given by:

(10) $$\begin{array}{c} x_{\mathrm{FD}}^{\left(i\right)}=\left(C_{1}\left(x_{1}^{\left(i\right)}\right),\;C_{2}\left(x_{2}^{\left(i\right)}\right),\;\ldots ,\;C_{J}\left(x_{J}^{\left(i\right)}\right)\right) \end{array}$$

where $x_{\mathrm{FD}}^{\left(i\right)}$ is a feature vector of *J* composed of discretized feature indices, and serves as the input to subsequent models.

Through the aforementioned procedure, continuous features can be accurately transformed into categorical interval representations, thereby achieving a desirable balance between computational efficiency, interpretability, and classification performance. Unlike traditional unsupervised binning methods, the proposed approach leverages a supervised CART-based discretization strategy, which adaptively partitions feature values based on label information. The use of hyperparameters $D_{j}$ (maximum tree depth) and $L_{j}$ (maximum number of leaf nodes) imposes strict constraints on partitioning complexity, effectively mitigating the risk of overfitting, as shown in Algorithm 1. This discretization procedure is particularly well suited for datasets characterized by high skewness or severe class imbalance, where conventional methods often struggle to capture meaningful boundaries.
**Algorithm 1** CART-based Feature Discretization**Input:** Continuous feature column $X_{j}={\left\{x_{j}^{\left(i\right)}\right\}}_{i=1}^{N}$, labels $y={\left\{y_{i}\right\}}_{i=1}^{N}$, max tree depth $D_{j}$, max leaf nodes $L_{j}$.
**Output:** Discretized feature indices $Z_{j}={\left\{z_{j}^{\left(i\right)}\right\}}_{i=1}^{N}$.
1.  Initialize CART tree structure $T_{j}$2.  Set $T_{j}.\max\_\mathrm{depth}\leftarrow D_{j}$3.  Set $T_{j}.\max\_\mathrm{leaf}\_\mathrm{nodes}\leftarrow L_{j}$4.  Train $T_{j}$ using paired samples $\left(x_{j}^{\left(i\right)},y_{i}\right)$5.  Extract unique splitting thresholds: $\theta_{j}\leftarrow \mathrm{ExtractThresholds}\left(T_{j}\right)$6.  Sort $\theta_{j}$ in ascending order7.  $M\leftarrow \mathrm{length}(\theta_{j})$, $K_{j}\leftarrow M+1$8.  Set $\theta_{j}^{\left(0\right)}\leftarrow -\infty$, $\theta_{j}^{\left(K_{j}\right)}\leftarrow +\infty$9.  Define $\theta_{j}^{\mathrm{ext}}=\left\{\theta_{j}^{\left(0\right)},\ldots ,\theta_{j}^{\left(K_{j}\right)}\right\}$10.   Initialize $Z_{j}$ of size *N*11.   **for** i=1 to *N*12.    **for** k=1 to $K_{j}$13.        **if** $\theta_{j}^{\left(k-1\right)}<x_{j}^{\left(i\right)}\leq \theta_{j}^{\left(k\right)}$ then14.            $z_{j}^{\left(i\right)}\leftarrow k$15.       **break**16.   **return** $Z_{j}$


### 3.3. Base Models Construction

Having obtained the discretized feature representations, the next stage involves training diverse base models tailored to these heterogeneous feature types. In the FADEL framework, the core objective of the base model construction phase is to perform dedicated modeling for different types of input, namely, continuous variables and discretized features. This strategy aims to maximize the sensitivity of each learner to its designated feature representation. All input data are derived from the feature space $X\subseteq R^{d}$, with the label space defined as Y={0,1}. After preprocessing, the dataset is partitioned according to feature discretization decisions into non-discretized training and testing sets, denoted as $\left\{ND_{tr},ND_{te}\right\}$, and discretized training and testing sets, denoted as $\left\{FD_{tr},FD_{te}\right\}$. These subsets are then projected through distinct feature mapping functions, denoted by $\phi_{ND}$ and $\phi_{FD}$, respectively. Accordingly, for each sample $x_{i}\in X$, the feature representations are given by:

For each sample $x_{i}\in X$, the feature mappings are defined as:

(11) $$\begin{array}{c} x_{i}^{ND}=\phi_{ND}\left(x_{i}\right)\in R^{d},\;x_{i}^{FD}=\phi_{FD}\left(x_{i}\right)\in Z^{d^{\prime }} \end{array}$$

where $Z^{d^{\prime }}$ represents the interval-encoded feature space obtained through supervised CART-based discretization. The four base models are each modeled as a mapping function $H_{m}$, where m∈{1,2,3,4}. Specifically, XGBoost and LightGBM correspond to $H_{1}$ and $H_{2}$, taking non-discretized features as input, while CatBoost and AdaBoost correspond to $H_{3}$ and $H_{4}$, which use discretized features. Thus, the general mapping function for all base models is defined as:

(12) $$\begin{array}{c} H_{m}:R^{d}\cup Z^{d^{\prime }}\to R \end{array}$$

The training objective of each base model is to minimize the empirical risk ${\hat{R}}_{m}$. Let ${\hat{y}}_{i}=\sigma \left(s_{m}^{\left(i\right)}\right)$ be the predicted output, where $\sigma \left(z\right)=\frac{1}{1+\exp(-z)}$ is the sigmoid function. Then:

(13) $$\begin{array}{c} {\hat{R}}_{m}=\frac{1}{N}\sum_{i=1}^{N}L\left(y_{i},{\hat{y}}_{i}\right) \end{array}$$

with the loss function defined as the binary cross-entropy:

(14) $$\begin{array}{c} L\left(y_{i},{\hat{y}}_{i}\right)=-\left[y_{i}\log\left({\hat{y}}_{i}\right)+\left(1-y_{i}\right)\log\left(1-{\hat{y}}_{i}\right)\right] \end{array}$$

Substituting the loss into the risk, we obtain:

(15) $$\begin{array}{c} {\hat{R}}_{m}=-\frac{1}{N}\sum_{i=1}^{N}\left[y_{i}\log\left({\hat{y}}_{i}\right)+\left(1-y_{i}\right)\log\left(1-{\hat{y}}_{i}\right)\right] \end{array}$$

Each base learner is optimized via:

(16) $$\begin{array}{c} {\hat{h}}_{m}=\arg\min_{h\in H_{m}}{\hat{R}}_{m} \end{array}$$

Specifically, for $H_{1}$ (XGBoost), the model updates the cumulative prediction score of sample *i* at iteration *t* as:

(17) $$\begin{array}{c} s_{m}^{\left(i,t\right)}=s_{m}^{\left(i,t-1\right)}+f_{t}\left(x_{i}^{ND}\right) \end{array}$$

Let $f_{t}$ denote the structure function of the *t*-th decision tree. The Taylor expansion of the log-loss function at $s_{m}^{\left(i,t\right)}$ can be approximated as:

(18) $$\begin{array}{c} L\left(y_{i},\sigma \left(s_{m}^{\left(i,t\right)}\right)\right)\approx L\left(y_{i},\sigma \left(s_{m}^{\left(i,t-1\right)}\right)\right)+g_{i}f_{t}\left(x_{i}^{ND}\right)+\frac{1}{2}h_{i}{\left(f_{t}\left(x_{i}^{ND}\right)\right)}^{2} \end{array}$$

where the first- and second-order derivatives of the loss function with respect to *s* are given by:

(19) $$\begin{array}{c} g_{i}=\frac{\partial L(y_{i},\sigma \left(s\right))}{\partial s}{|}_{s=s_{m}^{\left(i,t-1\right)}},\;h_{i}=\frac{\partial^{2}L\left(y_{i},\sigma \left(s\right)\right)}{\partial s^{2}}{|}_{s=s_{m}^{\left(i,t-1\right)}} \end{array}$$

The corresponding gain from a candidate split is computed as:

(20) $$\begin{array}{c} \mathrm{Gain}=\frac{1}{2}\left[\frac{{\left(\sum_{i\in L}g_{i}\right)}^{2}}{\sum_{i\in L}h_{i}+\lambda }+\frac{{\left(\sum_{i\in R}g_{i}\right)}^{2}}{\sum_{i\in R}h_{i}+\lambda }-\frac{{\left(\sum_{i\in L\cup R}g_{i}\right)}^{2}}{\sum_{i\in L\cup R}h_{i}+\lambda }\right]-\gamma \end{array}$$

where λ is the L2 regularization parameter and γ is the regularization term penalizing excessive splits. LightGBM adopts a histogram-based gradient splitting strategy, which first bins feature values to reduce the cost of sorting and partitioning. In each iteration, the prediction is accumulated similarly as in Equation (17). However, the gain is calculated from histogram statistics:

(21) $$\begin{array}{c} \mathrm{Gain}=\frac{1}{2}\left[\frac{G_{L}^{2}}{H_{L}+\lambda }+\frac{G_{R}^{2}}{H_{R}+\lambda }-\frac{{\left(G_{L}+G_{R}\right)}^{2}}{H_{L}+H_{R}+\lambda }\right]-\gamma \end{array}$$

where $G_{L}$ and $G_{R}$ are the sums of gradients, and $H_{L}$ and $H_{R}$ are the sums of Hessians for the left and right child nodes, respectively. For the CatBoost model, each discretized feature is sorted, and the target encoding (TE) is defined as:

(22) $$\begin{array}{c} \mathrm{TE}\left(x_{j}^{FD}\right)=\frac{\sum_{k=1}^{i-1}1\left\{x_{k,j}^{FD}=x_{i,j}^{FD}\right\}\cdot y_{k}+a\cdot p}{\sum_{k=1}^{i-1}1\left\{x_{k,j}^{FD}=x_{i,j}^{FD}\right\}+a} \end{array}$$

where *a* is a smoothing parameter and *p* is the prior probability. As for the AdaBoost model, sample weights are updated iteratively. After training the weak learner $h_{t}$ at iteration *t*, the classification error is computed as:

(23) $$\begin{array}{c} \epsilon_{t}=\sum_{i=1}^{N}w_{i}^{\left(t\right)}\cdot 1\left\{h_{t}\left(x_{i}^{FD}\right)\neq y_{i}\right\} \end{array}$$

The weight update rule is then defined by:

(24) $$\begin{array}{c} \alpha_{t}=\frac{1}{2}\log\left(\frac{1-\epsilon_{t}}{\epsilon_{t}}\right),\;w_{i}^{\left(t+1\right)}=w_{i}^{\left(t\right)}\exp\left(-\alpha_{t}\cdot y_{i}\cdot h_{t}\left(x_{i}^{FD}\right)\right) \end{array}$$

Finally, the outputs of all weak learners are aggregated to form the ensemble score. Once all base learners are trained, for any test sample $x_{*}$, the output score vector is:

(25) $$\begin{array}{c} s_{*}=\left[s_{1}^{\left(*\right)},\;s_{2}^{\left(*\right)},\;s_{3}^{\left(*\right)},\;s_{4}^{\left(*\right)}\right] \end{array}$$

After applying the sigmoid transformation to each base model’s score, the resulting probability outputs form a feature vector, which is subsequently used as input to the random forest meta-model:

(26) $$\begin{array}{c} v_{*}={\left[{\hat{p}}_{1}^{\left(*\right)},\;{\hat{p}}_{2}^{\left(*\right)},\;{\hat{p}}_{3}^{\left(*\right)},\;{\hat{p}}_{4}^{\left(*\right)}\right]}^{⊤} \end{array}$$

In this study, the selection of the base model adheres to the principle of maximizing diversity in ensemble learning, aiming to construct a highly complementary model in terms of bias–variance trade-off and decision boundary structure. This strategy leverages algorithmic complementarity to enhance the overall predictive capacity of the model. Accordingly, we integrate four distinct learners: XGBoost and LightGBM, which are gradient boosting algorithms known for their ability to model complex non-linear relationships and reduce bias; AdaBoost, which focuses on enhancing the recognition of hard-to-classify instances; and CatBoost, which demonstrates robustness when handling high-cardinality categorical features.

To further strengthen model diversity, we exploit heterogeneity in both feature representation and decision boundary formation. XGBoost and LightGBM are trained on original continuous features, enabling them to learn flexible and precise non-linear decision boundaries. In contrast, AdaBoost and CatBoost operate on features that have undergone supervised discretization, resulting in step-wise decision boundaries constrained by predefined intervals—structures that tend to be more stable and particularly effective for capturing patterns in minority classes. Through this two-level diversity design—spanning both algorithmic and feature representation dimensions—the FADEL framework is capable of exploring the feature space from multiple perspectives. By effectively combining low-bias models with highly stable learners, FADEL demonstrates superior sensitivity to minority classes and outstanding generalization performance, especially under scenarios of extreme class imbalance, as shown in Algorithm 2.
**Algorithm 2** FADEL Base Models Construction**Input:** Feature space X, labels Y, dataset $D={\left\{\left(x_{i},y_{i}\right)\right\}}_{i=1}^{N}$
**Output:** Trained models ${\left\{{\hat{h}}_{m}\right\}}_{m=1}^{4}$
1.  Partition *D* into $ND_{tr},ND_{te}$ and $FD_{tr},FD_{te}$2.  **For** i=1 to *N*:3.      Compute $x_{i}^{ND}=\phi^{ND}\left(x_{i}\right)\in R^{d}$4.      Compute $x_{i}^{FD}=\phi^{FD}\left(x_{i}\right)\in Z^{d^{\prime }}$5.  Define mappings: $H_{m}:R^{d}\cup Z^{d^{\prime }}\to R,\;m=1,\ldots ,4$6.  **For** m=1 to 4:7.      Minimize empirical risk: ${\hat{R}}_{m}=\frac{1}{N}\sum_{i=1}^{N}\left\{-\left[y_{i}\log{\hat{y}}_{i}+\left(1-y_{i}\right)\log\left(1-{\hat{y}}_{i}\right)\right]\right\}$8.  **For** m=1,2 (XGBoost, LightGBM):9.      Compute gradients $g_{i}$ and Hessians $h_{i}$10.        Update score: $s_{m}^{\left(i,t\right)}=s_{m}^{\left(i,t-1\right)}+f_{t}\left(x_{i}^{ND}\right)$11.           Compute split gain (21)12.   **For** m=3 (CatBoost):13.     Compute target encoding (22)14.   **For** m=4 (AdaBoost):15.     Update weights: $w_{i}^{\left(t+1\right)}=w_{i}^{\left(t\right)}\exp\left(-\alpha_{t}\;y_{i}\;h_{t}\left(x_{i}^{FD}\right)\right)$16.   **return** ${\left\{{\hat{h}}_{m}\right\}}_{m=1}^{4}$


### 3.4. Ensemble Learning-Based Prediction

Leveraging the prediction outputs of these heterogeneous base models, the final stage of the FADEL framework employs ensemble learning to produce robust predictions. Following the completion of feature discretization and base model training, the FADEL framework proceeds to integrate the prediction scores generated by each base learner on a given test sample. This is accomplished via a second-level meta-model—specifically, a Random Forest—that produces the final prediction. This stage consists of two major steps: (1) generation of the output score vector from the base models, and (2) decision fusion by the meta-model.

For a given test instance $x_{*}$, let $H_{m}$ denote the *m*-th base model. Its raw score output is defined as:

(27) $$\begin{array}{c} s_{m}\left(*\right)=H_{m}\left(x_{*}\right) \end{array}$$

Each score is transformed into a probability through the sigmoid function:

(28) $$\begin{array}{c} {\hat{p}}_{m}^{\left(*\right)}=\sigma \left(s_{m}\left(*\right)\right)=\frac{1}{1+\exp(-s_{m}\left(*\right))} \end{array}$$

The resulting probability vector $v_{*}={\left[{\hat{p}}_{1}^{\left(*\right)},\;{\hat{p}}_{2}^{\left(*\right)},\;{\hat{p}}_{3}^{\left(*\right)},\;{\hat{p}}_{4}^{\left(*\right)}\right]}^{⊤}$ serves as the input to the Random Forest meta-model.

Assume the Random Forest consists of *T* decision trees. The decision function of the *t*-th tree is:

(29) $$\begin{array}{c} T_{t}:R^{4}\to Y \end{array}$$

Each tree partitions the input space into a set of leaf regions $R_{t,j}$, and stores in each leaf a probability vector:

(30) $$\begin{array}{c} q_{t,j}=\left[q_{t,j}\left(y=1\right),\;q_{t,j}\left(y=0\right)\right] \end{array}$$

For input $v_{*}$, the prediction of the *t*-th tree is:

(31) $$\begin{array}{c} {\hat{y}}_{t}\left(*\right)=\arg\max_{c\in Y}q_{t,j}\left(y=c\right),\;\mathrm{where}\;v_{*}\in R_{t,j} \end{array}$$

The final output is determined by majority voting:

(32) $$\hat{y}\left(*\right)=\mathrm{mode}\left\{{\hat{y}}_{1}^{\left(*\right)},\;{\hat{y}}_{2}^{\left(*\right)},\;\ldots ,\;{\hat{y}}_{T}^{\left(*\right)}\right\}$$

Random Forests, serving as the meta-model, not only flexibly integrate the outputs of individual base models but also exhibit multiple advantages. First, by leveraging feature subsampling and bootstrap aggregation (bagging), they can effectively enhance the stability of minority class recognition and mitigate the risk of overfitting caused by bias toward individual features. Second, when aggregating predictions from heterogeneous feature types, Random Forests are capable of suppressing the influence of high-variance weak learners while improving the overall model consistency and generalization ability. Furthermore, Random Forests provide feature importance assessments derived from each decision tree’s contribution to the final prediction, thereby enhancing model interpretability and transparency. The predicted label $\hat{y}$ in Algorithm 3 is determined via majority voting over the predictions of all trees. The detailed procedure is described in Algorithm 3, where the final output is denoted as $\hat{y}\left(*\right)$.
**Algorithm 3** FADEL Ensemble Learning-based Prediction**Input:** Test sample $x_{*}$; trained base models ${\left\{H_{m}\right\}}_{m=1}^{4}$; Random Forest meta-model RF with *T* trees
**Output:** Final prediction $\hat{y}\left(x_{*}\right)$
1.  Initialize probability vector *v* of length 42.  **For** m=1 to 4:3.      Compute raw score: $s_{m}\leftarrow H_{m}\left(x_{*}\right)$4.          Compute probability: $p_{m}\leftarrow \sigma \left(s_{m}\right)=\frac{1}{1+\exp(-s_{m})}$5.      $v\left[m\right]\leftarrow p_{m}$6.  Initialize predictions array ${\left\{{\hat{y}}_{t}\right\}}_{t=1}^{T}$7.  **For** t=1 to *T*:8.      Identify leaf region $R_{t,j}$ containing *v*9.          Retrieve probability vector: $q_{t,j}$10.        Compute prediction: ${\hat{y}}_{t}\left(x_{*}\right)=\arg\max_{c\in Y}q_{t,j}\left(y=c\right)$11.     ${\hat{y}}_{t}\left[t\right]\leftarrow {\hat{y}}_{t}^{\left(*\right)}$12.   Aggregate final prediction by majority voting: $\hat{y}\left(*\right)=\mathrm{mode}\left\{{\hat{y}}_{1}^{\left(*\right)},{\hat{y}}_{2}^{\left(*\right)},\ldots ,{\hat{y}}_{T}^{\left(*\right)}\right\}$13.   **return** $\hat{y}\left(x_{*}\right)$


Based on the aforementioned analysis, the FADEL framework constructs a highly flexible and robust ensemble decision model by leveraging the probabilistic output diversity of four base models during the ensemble prediction stage and employing a non-linear integration mechanism via a Random Forest meta-model. Consequently, the proposed method in this study can effectively improve the sensitivity, specificity, and overall generalization performance for minority classes, even under conditions without any data-level balancing.

## 4. Experimental Study

The proposed FADEL framework was evaluated using a large-scale Kawasaki Disease (KD) dataset collected from Kaohsiung Chang Gung Memorial Hospital (CGMH), comprising a total of 79,400 electronic medical records. To rigorously assess the predictive performance of the model under an imbalanced classification scenario, the original distribution of negative and positive cases was preserved. The dataset was partitioned into training and testing sets using stratified sampling with an 80:20 ratio. The training set consisted of 63,520 samples, including 62,606 negative cases (febrile patients without KD) and 914 positive cases (patients diagnosed with KD), resulting in an imbalance ratio of 68.5%. The testing set contained 16,794 samples, with 16,566 negative and 228 positive cases, yielding an imbalance ratio of 72.7%. Please refer to Table 2 for detailed statistics.

To further validate the generalizability of the FADEL model, an external validation set comprising 1582 samples was obtained from Kaohsiung Medical University Chung-Ho Memorial Hospital(KMUH). This dataset includes 1520 negative cases and 62 positive cases, resulting in an imbalance ratio of 24.5%. The experimental design consists of two major components: (1) Experiment on the analysis of non-discretized versus feature discretization across the CGMH test set and KMUH validation set; and (2) comparing the predictive performance of ensemble models trained on datasets augmented through data-level balancing techniques, thereby demonstrating that the FADEL framework can achieve superior performance under imbalanced conditions without relying on data synthesis or balancing methods. Additionally, the model exhibits stable and consistent performance across different clinical institutions. All dataset partitions were stratified to maintain class proportions consistent with the original population distribution, thus minimizing sampling bias.

***Statistical Analysis***

Independent-sample *t*-tests were conducted to calculate confidence intervals (CIs) and *p*-values in order to quantify the statistical significance of performance differences among the evaluated methods. All statistical tests were two-tailed, with a significance level set at 0.05. Relevant statistical outcomes are reported in Table 2. This research framework employs real-world imbalanced KD datasets from both Kaohsiung Chang Gung Memorial Hospital and Kaohsiung Medical University Chung-Ho Memorial Hospital. The study was approved by the Chang Gung Medical Foundation Institutional Review Board (IRB Nos. 202202165B0 and 202202165B0C501). A waiver of informed consent was granted as all collected data were de-identified, ensuring that research personnel could not trace any participant’s identity. Data access and collection commenced on 7 February 2023, following IRB approval. All data acquisition procedures and related documentation were thoroughly reviewed and approved by the IRB.

***Assessment Measures***

To comprehensively evaluate the model’s recognition ability under imbalanced data conditions, multiple metrics were adopted, including F1-score, G-mean, recall (sensitivity), and specificity [29,46,47]. The F1-score balances precision and recall, while G-mean assesses the model’s capability to handle imbalanced class distributions. Sensitivity and specificity respectively reflect the prediction accuracy for the minority and majority classes.

***Base Models***

The proposed FADEL framework incorporates four types of base models: XGBoost, LightGBM, CatBoost, and AdaBoost. XGBoost and LightGBM directly utilize raw continuous features, enabling them to capture complex non-linear patterns. In contrast, CatBoost and AdaBoost are applied to supervised discretized features, which enhance their ability to recognize minority classes. Each base model constructs multiple decision trees during the training phase to reinforce generalization capability. Detailed model configurations are summarized in Table 3.

### 4.1. Experiment on the Analysis of Non-Discretized Versus Feature Discretization Across the CGMH Test Set and KMUH Validation Set

As shown in Table 4, models evaluated on the Kaohsiung Chang Gung testing dataset without feature discretization exhibited limited overall performance. The sensitivity of XGBoost, LightGBM, and the traditional stacking model ranged only from 80.8% to 84.7%, with the highest F1-score reaching merely 70%. These results indicate that even ensemble methods failed to sufficiently capture minority class characteristics in the absence of feature discretization.

**Figure 2:**
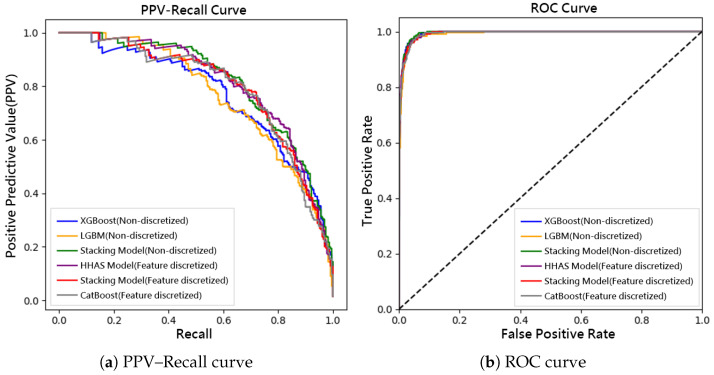
Comparison of model performance on the Kaohsiung Chang Gung Memorial Hospital Kawasaki Disease testing set: (**a**) Precision–Recall (PPV–Recall) curves and (**b**) Receiver Operating Characteristic (ROC) curves for all evaluated models. The dashed diagonal line in the ROC plot represents the performance of a random classifier.

In contrast, applying supervised feature discretization led to significant improvements across all evaluation metrics. Notably, the proposed FADEL model achieved a high recall rate of 90.8%, while attaining an F1-score of 61.7% and a G-mean of 94.5%, demonstrating superior sensitivity to rare cases. These findings confirm that supervised discretization clarifies minority class boundaries and enhances adaptability in imbalanced scenarios. Figure 2 Comparison of model performance on the Kaohsiung Chang Gung Memorial Hospital KD testing set: (2a) Precision–Recall (PPV–Recall) curves and (2b) Receiver Operating Characteristic (ROC) curves for all evaluated models.

**Figure 3:**
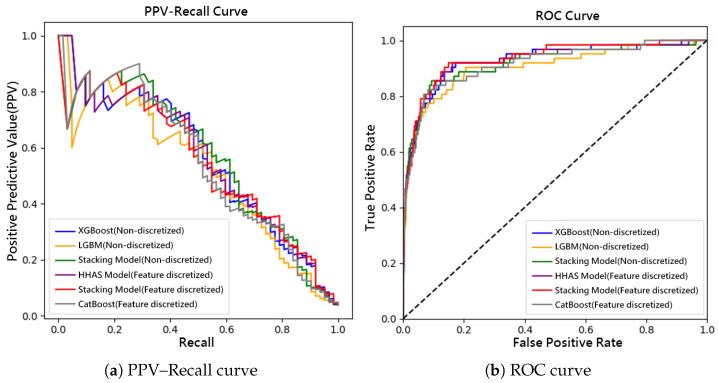
Comparison of model performance on the Kaohsiung Medical University Chung-Ho Memorial Hospital Kawasaki Disease testing set: (**a**) Precision–Recall (PPV–Recall) curves and (**b**) Receiver Operating Characteristic (ROC) curves for all evaluated models. The dashed diagonal line in the ROC plot represents the performance of a random classifier.

In the external validation dataset from Kaohsiung Medical University Chung-Ho Memorial Hospital, models without feature discretization demonstrated notably poor performance in identifying the minority class. The F1-scores of XGBoost and LightGBM were merely 31.6% and 31.3%, respectively, while the traditional stacking model achieved a slightly higher F1-score of 42.4%, which still falls below acceptable standards. After applying feature discretization, CatBoost achieved a sensitivity of 85.5% despite a modest F1-score of 29.7%. In contrast, the FADEL model maintained strong stability with a recall of 91.9% and a G-mean of 86.7%, indicating superior generalization and consistency. It is worth noting that some models, such as the stacking model, experienced a drop in F1-score on external data after discretization, likely due to distributional shifts across institutions. Nevertheless, FADEL consistently retained a comparative advantage. Figure 3 presents the (3a) PPV–Recall and (3b) ROC curves of all models on the KMUH test set.

A comparative summary of the experimental results reveals that models without discretization consistently underperformed in both sensitivity and F1-score across the two datasets, particularly on the KMUH external validation set, where minority class recognition was markedly deficient. This suggests that relying solely on raw continuous variables is susceptible to the adverse effects of skewed distributions and outliers, leading to degraded predictive performance. In contrast, the proposed FADEL model, by leveraging supervised discretization and heterogeneous ensemble learning, exhibited robust and superior performance across both datasets. The FADEL model achieved 90.8% sensitivity and the highest F1-score on the CGMH test set, and maintained 91.9% recall and 86.7% G-mean on the KMUH validation set, demonstrating strong practical applicability and generalization capability.

Building on these results, further analysis was conducted to examine the impact of feature discretization on data distribution, particularly in terms of skewness correction. The Shapiro–Wilk normality test was applied to all continuous features in both the CGMH and KMUH datasets to assess deviations from normal distribution. The results confirmed that the overall data exhibited significant non-normality. According to the standard interpretation of skewness, features with |Skewness|>1 are considered highly skewed, which may adversely affect model parameter estimation and learning stability. As shown in Table 5, several features—such as AST, ALT, and CRP—exhibited extreme positive skewness, with values of 114.53, 78.73, and 3.76, respectively. After applying supervised decision tree threshold-based discretization, the skewness of most features was substantially reduced (e.g., AST from 114.53 to 0.84; CRP from 3.76 to 1.62), indicating that this method is effective in mitigating skewness and stabilizing data distributions.

These findings demonstrate that discretization not only suppresses the influence of extreme values on parameter learning, but also segments highly skewed features into more uniform intervals. This facilitates the model’s ability to capture non-linear patterns and distributional trends. Additionally, feature discretization simplifies variable structures and reduces the impact of noise and outliers, thereby improving training efficiency and prediction stability. For highly imbalanced classification tasks, this strategy further accentuates the distribution characteristics of minority classes, enabling the model to maintain strong sensitivity and overall performance across diverse data sources.

### 4.2. Comparison of Predictive Performance of Ensemble Models Trained with Data-Level Augmentation Techniques

To substantiate the advantages of the proposed FADEL framework, this section presents a comparative analysis with traditional ensemble learning methods reliant on data-level balancing. The evaluation focuses on their effectiveness in identifying minority class instances under highly imbalanced conditions. For data augmentation, we employ the Conditional Tabular Generative Adversarial Network (CTGAN), a widely adopted technique capable of modeling continuous and categorical distributions while preserving the original dataset’s statistical properties and latent feature correlations. CTGAN is used to increase the number of minority class samples in the training set to match the majority class, establishing a fair basis for comparison.

As shown in Table 6, within the CGMH test set experiment, CTGAN augmented the minority class from 1230 to 78,170 samples to achieve balance. In this setting, the non-discretized XGBoost model attained a sensitivity of 80.8% and an F1-score of 69.7%, indicating that synthetic data generation enhances minority class detection. However, the traditional Stacking model, despite achieving a high sensitivity of 96.1%, yielded an F1-score of 47.1%, reflecting a recall-precision trade-off. In contrast, the FADEL model, leveraging supervised feature discretization, achieved 95.6% sensitivity, an F1-score of 47.8%, and a G-mean of 96.2%, demonstrating both stability and robustness. Notably, while CTGAN-based augmentation improved recall and G-mean in certain cases, it reduced specificity; for instance, the Stacking model’s specificity dropped to 95.5%, lower than its performance under unbalanced training conditions.

A parallel experiment using the KMUH external validation set involved augmenting the minority class from 62 to 1520 samples using CTGAN. The non-discretized XGBoost model maintained a sensitivity of 80.6% and an F1-score of 40.2%, consistent with the CGMH results. However, the traditional Stacking model, despite a sensitivity of 85.5%, achieved an F1-score of only 32.8%, indicating that improved recall did not enhance overall classification performance. Under discretized conditions, its F1-score further declined to 19.5%, revealing instability when synthetic data are applied across institutions. Conversely, the FADEL model retained a sensitivity of 90.3%, an F1-score of 27.5%, and a G-mean of 85.6%, reflecting stable generalization. Despite the modest F1-score, FADEL consistently prioritizes high sensitivity, a critical property in clinical applications where minimizing false negatives is paramount.

In summary, while CTGAN-based augmentation enhances recall under extreme imbalance, it introduces significant computational overhead and often degrades performance due to distributional distortion and suboptimal boundary alignment. In contrast, FADEL eliminates the need for synthetic data by employing type-aware feature routing: raw continuous and supervised discretized features are allocated to optimal classifiers, enabling robust learning of non-linear and interval-based patterns. The experimental results confirm that FADEL achieves over 90% recall and F1-scores comparable to or surpassing traditional methods without data augmentation. These findings underscore FADEL’s superior computational efficiency, predictive robustness, and cross-institutional generalizability, establishing it as a groundbreaking solution for addressing extreme class imbalance challenges in clinical and other high-stakes applications.

## 5. Discussion

The proposed FADEL framework eliminates the need for any data-level augmentation techniques (such as SMOTE or CTGAN-generated samples) while still demonstrating superior predictive performance under highly imbalanced conditions. FADEL operates at the algorithmic level by leveraging the heterogeneity between continuous and discretized features, strategically integrating the complementary strengths of diverse base models. This design effectively avoids the information loss and noise typically introduced by feature homogenization or synthetic data generation, and also mitigates potential security risks such as data poisoning.

While recent studies in ensemble learning have predominantly focused on refining model architectures and fusion strategies, limited attention has been given to the structural representation of input data. This study provides empirical evidence that the manner in which data are represented and routed—specifically, the type-aware integration of raw continuous and discretized features—can significantly influence predictive performance under highly imbalanced conditions. These findings strongly support the notion that feature augmentation not only outperforms traditional data augmentation in addressing class imbalance, but also mitigates the risks associated with synthetic data generation and enhances overall model security.

Specifically, XGBoost and LightGBM, as gradient boosting algorithms, are well suited for capturing the non-linear boundaries of continuous variables. CatBoost natively supports categorical feature encoding, mitigating risks associated with dimensionality explosion from conventional one-hot encoding. AdaBoost, through its ensemble of weak learners, is particularly adept at handling discrete features and concentrating learning efforts on misclassified or borderline instances, thereby enhancing minority class recognition. These four models exhibit high complementarity in terms of feature-type sensitivity and error-correction strategies, forming a robust and diverse ensemble foundation for FADEL.

**Figure 4:**
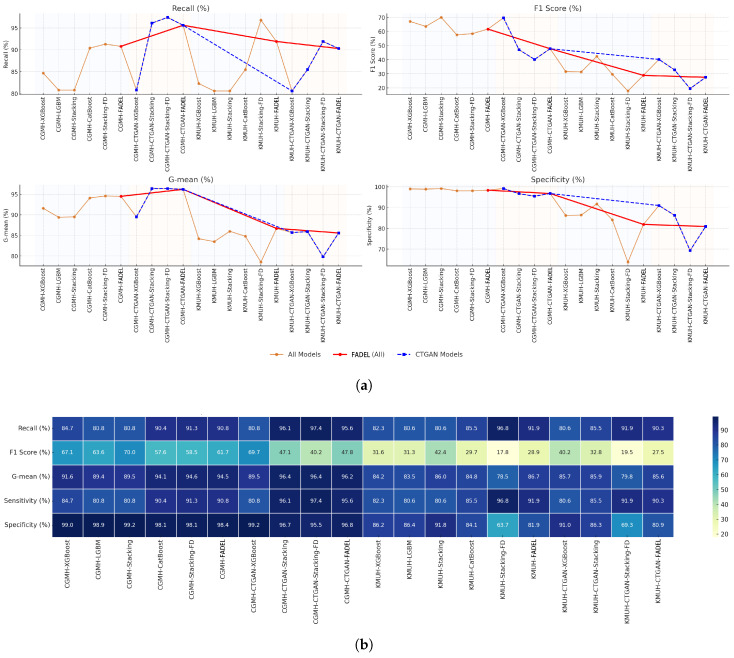
Comparative analysis of multiple models with the FADEL framework and data augmentation strategies on Kawasaki Disease datasets from Chang Gung Memorial Hospital (CGMH) and Kaohsiung Medical University Chung-Ho Memorial Hospital (KMUH). (**a**) Line plot comparison of FADEL and CTGAN-augmented models across key performance metrics (recall, F1-score, G-mean, and specificity), highlighting FADEL superiority and consistent performance in CGMH and KMUH datasets. (**b**) Comprehensive heatmap comparison of model performance across CGMH and KMUH datasets, with and without CTGAN-based data augmentation. Metrics include recall, F1-score, G-mean, sensitivity, and specificity across 12 model variants.

As shown in Figure 4a,b, FADEL achieves a recall rate of 90.8%, F1-score of 61.7%, and G-mean of 94.5% on the CGMH test set, outperforming both XGBoost with CTGAN augmentation (F1-score: 69.7%) and the traditional stacking model (F1-score: only 47.1%). Furthermore, on the KMUH validation set, FADEL maintains a recall of 91.9% and a G-mean of 86.7%, whereas the CTGAN-Stacking model yields an F1-score of only 19.5%, demonstrating FADEL’s strong generalizability across institutions.

The comparative results presented in Table 7 underscore the superior predictive performance of the proposed FADEL framework relative to existing methodologies for Kawasaki Disease classification. Unlike previous models that rely on logistic regression or deep neural network architectures, FADEL integrates feature-type awareness into an ensemble learning paradigm, achieving concurrently high sensitivity (91%) and specificity (98%), with a resulting G-mean of 95%—a robust indicator of balanced performance. Remarkably, this level of effectiveness is achieved without the use of any data-level augmentation techniques, such as SMOTE or GAN-based oversampling. In addition to its predictive accuracy, FADEL features a lightweight and computationally efficient design, making it highly suitable for deployment in real-world clinical settings. Collectively, these findings highlight FADEL’s potential as a scalable, generalizable, and resource-efficient solution for addressing extreme class imbalance in classification tasks.

## 6. Limitations and Future Work

Despite the superior performance demonstrated by the proposed FADEL framework, several limitations remain. First, under extreme class imbalance conditions, if the number of minority class samples is excessively sparse, the supervised decision tree used for feature discretization may lack sufficient information to support stable interval partitioning. This may result in threshold learning bias or over-segmentation, which could compromise model stability or even lead to training failures. Second, the current evaluation of FADEL is based on clinical datasets from Kawasaki Disease (KD) patients collected from two major hospitals in Taiwan. Future work should extend validation to other medical domains or datasets with higher heterogeneity to ensure broader applicability and improved generalizability.

Moreover, FADEL currently employs Random Forest as the meta-model by default, without exploring more advanced fusion strategies, such as attention-based weighting mechanisms or deep ensemble architectures. Future research can optimize FADEL along two directions: (1) In terms of feature augmentation (feature discretization), the incorporation of strategies such as equal-width binning, equal-frequency binning, dynamic interval learning, or entropy-based splitting may enhance segmentation stability and classification accuracy in extremely sparse data scenarios. Additionally, we suggest investigating a dual-input design in which both the raw continuous features and their discretized counterparts are simultaneously fed into the four base models, resulting in eight output predictions. The ensemble of these outputs may further improve the overall predictive performance. (2) Advanced deep learning architectures such as TabNet and Transformers can be integrated to strengthen the modeling of complex decision boundaries. FADEL can also be extended to highly imbalanced domains such as rare disease diagnosis, financial fraud detection, and anomaly detection, thereby validating its generalization potential and practical value when applied to heterogeneous and extremely skewed datasets.

## 7. Conclusions

The proposed FADEL framework addresses the challenge of extreme class imbalance without relying on data-level balancing or data augmentation techniques such as SMOTE or GAN-based sample generation. FADEL strategically routes continuous and discrete features into base models that are most sensitive to their respective feature types: XGBoost and LightGBM receive raw continuous variables to leverage their strong non-linear boundary-fitting capabilities, while CatBoost and AdaBoost are paired with supervised discretized features to enhance learning over interval-based patterns. This design effectively prevents information loss caused by forced feature homogenization and reduces noise and artifacts introduced by synthetic augmentation. Experimental results demonstrate that FADEL achieves over 90% recall on both internal and external datasets without any form of sample generation, while also delivering excellent performance in terms of F1-score and G-mean.

To further enhance the model’s robustness, future research could focus on developing category-sensitive adaptive feature augmentation(discretization) techniques to better capture patterns in rare classes. Additionally, alternatives to the current Random Forest meta-model could be explored, such as attention-weighted or self-supervised fusion mechanisms, to improve information aggregation at the ensemble level. In summary, FADEL uses feature augmentation to offer a high-performing and generalizable solution for imbalanced classification problems, without the need for data-level augmentation, demonstrating both practical significance and scalable value in real-world applications.

## Figures and Tables

**Table 1:** Summary of major mathematical notations.

Notations	Mathematical Meanings	Notations	Mathematical Meanings
$FD_{tr}$	Feature-discretized training dataset	$FD_{te}$	Feature-discretized testing dataset
$ND_{tr}$	Non-discretized (original) training dataset	$ND_{te}$	Non-discretized (original) testing dataset
X	Input feature space $R^{d}$	Y	Label space {0,1}
D	Complete dataset ${\left\{\left(x_{i},y_{i}\right)\right\}}_{i=1}^{N}$	*T*	Number of trees in the Random Forest meta-model
$\phi_{ND}$	Non-discretized feature projection function	$\phi_{FD}$	Discretized feature projection function
$x^{ND}$	Non-discretized feature vector	$x^{FD}$	Discretized feature vector
$H_{m}$	Mapping function of the *m*-th base learner	$s_{m}$	Raw output score of the *m*-th base learner
$p_{m}$	Predicted probability of the *m*-th base learner	*p*	Vector of base learners’ predicted probabilities
$\hat{y}$	Final predicted label	$f_{t}$	Output value function of tree *t*
$R_{l}^{\left(t\right)}$	Region of the *l*-th leaf node in tree *t*	$\theta_{j}^{\left(k\right)}$	*k*-th threshold of the *j*-th feature
$K_{j}$	Number of intervals for discretizing the *j*-th feature	$C_{j}\left(x\right)$	Discretization mapping function
$I_{jk}$	*k*-th interval of the *j*-th feature	$L_{j}$	Max number of leaf nodes in CART for feature *j*
$D_{j}$	Max depth of the CART tree for feature *j*	$h_{i}$	Second-order Hessian of the loss function
$g_{i}$	First-order gradient of the loss function	γ	Split penalty term in XGBoost and LightGBM
λ	L2 regularization in XGBoost and LightGBM	$R_{m}$	Empirical risk of the *m*-th base learner
L(·)	Loss function (e.g., logistic loss)	$w_{it}$	AdaBoost weight of sample *i* at iteration *t*
$\alpha_{t}$	AdaBoost weight of the *t*-th weak learner	σ(·)	Sigmoid function

**Table 2:** Descriptive statistics of the Kawasaki Disease datasets from CGMH and KMUH.

	Kaohsiung Chang Gung Memorial Hospital (N = 79,400)				Kaohsiung Medical University Chung-Ho Memorial Hospital (N = 1582)			
Characteristics	Children with KD (N = 1230)	Febrile Controls (N = 78,170)	Odds Ratio (95% CI)	* p * -Value	Children with KD (N = 62)	Febrile Controls (N = 1520)	Odds Ratio (95% CI)	* p * -Value
**Birth, mean (SD)**								
Age, y	1.2 (1.2)	1.8 (1.6)	0.603 (0.563–0.646)	<0.001	2.4 (2.5)	3.1 (1.9)	0.832 (0.696–0.995)	0.002
**Gender, No. (%)**								
Male	746 (60.7%)	44,037 (56.3%)	0.555 (0.482–0.639)	0.002	40 (64.5%)	1020 (67.1%)	0.951 (0.504–1.797)	0.431
**Blood, mean (SD)**								
RBC, $10^{6}/$µL	4.3 (0.4)	4.5 (0.6)	0.197 (0.091–0.423)	<0.001	4.4 (0.5)	4.6 (0.5)	69.5 (1.66–2909.689)	0.042
WBC, $10^{3}/$µL	14.1 (5.2)	10.8 (5.9)	1.001 (0.988–1.014)	<0.001	13.6 (5.6)	10.1 (5.3)	0.947 (0.888–1.01)	<0.001
Hemoglobin, g/dL	11.1 (1.1)	12 (1.8)	1.44 (0.863–2.403)	<0.001	11.3 (1.2)	12.0 (1.1)	1.55 (0.898–2.805)	<0.001
Hematocrit, %	33.4 (3.1)	35.6 (4.9)	1.069 (0.883–1.295)	<0.001	34.2 (2.9)	35.8 (2.9)	0.057 (0.005–0.648)	<0.001
MCH, pg/cell	25.7 (2.3)	27.1 (3.5)	0.623 (0.541–0.719)	<0.001	25.7 (3.2)	26.5 (2.5)	2.258 (1.082–4.711)	0.007
MCHC, g/dL	33.3 (1.1)	33.6 (1.2)	0.954 (0.776–1.173)	<0.001	33.1 (1.3)	33.5 (1.1)	0.044 (0.003–0.58)	<0.001
RDW	13.4 (1.4)	14.1 (2.1)	0.665 (0.633–0.7)	<0.001	13.6 (1.4)	13.3 (1.7)	0.843 (0.655–1.084)	0.072
Platelets, $10^{3}/$µL	350.1 (125.8)	294.2 (125)	1.002 (1.001–1.003)	<0.001	324.6 (119.1)	263.7 (90.7)	1.004 (1.001–1.008)	<0.001
Neutrophils-segments, %	56.3 (15.4)	49.3 (19.9)	1.186 (1.147–1.227)	<0.001	58.2 (18.0)	57.9 (19.3)	0.981 (0.924–1.04)	0.9
Neutrophils-bands, %	1.6 (3.2)	0.7 (2.1)	1.232 (1.182–1.283)	<0.001	4.8 (7.2)	2.8 (6.2)	0.957 (0.887–1.034)	0.007
Lymphocyte, %	32.1 (13.9)	38.3 (18.3)	1.156 (1.116–1.196)	<0.001	26.3 (13.8)	29.5 (16.6)	0.972 (0.913–1.035)	0.114
Monocyte, %	6.8 (3.4)	8.4 (4.3)	1.039 (0.998–1.081)	<0.001	6.8 (3.3)	8.1 (3.6)	0.934 (0.844–1.034)	0.004
Eosinophils, %	2.5 (2.7)	1.8 (2.9)	1.325 (1.275–1.376)	<0.001	2.5 (2.7)	0.8 (1.6)	1.2 (1.071–1.345)	<0.001
Basophils, %	0.2 (0.4)	0.3 (0.4)	1.175 (1.026–1.347)	0.006	0.2 (0.4)	0.3 (0.4)	0.851 (0.394–1.836)	0.345
AST, U/L	77.9 (117.1)	51 (130.6)	0.997 (0.997–0.998)	<0.001	84.0 (102.0)	42.5 (31.9)	0.991 (0.986–0.997)	<0.001
ALT, U/L	76.0 (108.3)	34.6 (78.9)	1.005 (1.004–1.006)	<0.001	91.0 (127.8)	21.0 (24.6)	1.021 (1.013–1.029)	<0.001
CRP, mg/dL	74.9 (61.5)	20.1 (32.8)	1.015 (1.014–1.017)	<0.001	79.9 (49.5)	23.4 (34.8)	1.022 (1.016–1.028)	<0.001
**Urine, mean (SD)**								
WBC count in urine, count/hpf	54.3 (101.6)	49.9 (66.8)	0.992 (0.991–0.993)	0.023	11.3 (21.9)	4.5 (13.9)	0.986 (0.968–1.004)	<0.001
**Urine, No. (%)**								
Pyuria	526 (42.8%)	5416 (6.9%)	0.093 (0.079–0.108)	<0.001	22 (31.9%)	195 (10.2%)	2.604 (1.09–6.219)	<0.001

Descriptive Statistics of the Kaohsiung Chang Gung Memorial Hospital Test Dataset and Kaohsiung Medical University Chung-Ho Memorial Hospital Validation Dataset.

**Table 3:** Summary of hyperparameters and configuration settings.

Notation	Parameter Description	Value
**Feature-Discretization Parameters**		
$D_{j}$	Maximum depth of the CART tree for discretizing feature *j*	3
$L_{j}$	Maximum number of leaf nodes in the CART tree for feature *j*	8
Split Criterion	Criterion used to measure the quality of a split	Gini impurity
Minimum Samples per Leaf	Minimum number of samples required to be at a leaf node	5
Minimum Samples per Split	Minimum number of samples required to split an internal node	2
**Ensemble Learning Model Parameters**		
*Random Forest Meta-model*		
*T*	Number of trees in Random Forest meta-model	100
*XGBoost and LightGBM Base models*		
γ	Split penalty term in XGBoost and LightGBM	0
λ	L2 regularization parameter in XGBoost and LightGBM	1
*CatBoost Base model*		
$M_{\mathrm{CAT}}$	Number of boosting iterations in CatBoost	100
$\eta_{\mathrm{CAT}}$	Learning rate in CatBoost	0.1
$max\_depth_{\mathrm{CAT}}$	Maximum depth of trees in CatBoost	6
*AdaBoost Base model*		
$M_{\mathrm{ADA}}$	Number of estimators in AdaBoost	50
$\eta_{\mathrm{ADA}}$	Learning rate in AdaBoost	1

**Table 4:** Performance comparison across different models and discretization strategies on two Kawasaki Disease datasets from Chang Gung Memorial Hospital (CGMH) and Kaohsiung Medical University Hospital (KMUH).

Metric	Kaohsiung Chang Gung Memorial Hospital KD Test Set						Kaohsiung Medical University Chung-Ho Memorial Hospital KD Validation Set					
	Non-Discretized (Original Data)			Feature-Discretized (Feature Augmentation)			Non-Discretized (Original Data)			Feature-Discretized (Feature Augmentation)		
	XGBoost	LightGBM	Stacking Model	CatBoost	Stacking Model	FADEL Model	XGBoost	LightGBM	Stacking Model	CatBoost	Stacking Model	FADEL Model
Recall/Sensitivity (%)	84.7	80.8	80.8	90.4	91.3	90.8	82.3	80.6	80.6	85.5	96.8	91.9
F1 Score (%)	67.1	63.6	70.0	57.6	58.5	61.7	31.6	31.3	42.4	29.7	17.8	28.9
G-mean (%)	91.6	89.4	89.5	94.1	94.6	94.5	84.2	83.5	86.0	84.8	78.5	86.7
Specificity (%)	99.0	98.9	99.2	98.1	98.1	98.4	86.2	86.4	91.8	84.1	63.7	81.9

**Table 5:** Comparison of feature skewness between non-discretized and discretized representations in Kawasaki Disease datasets from the Chang Gung Memorial Hospital (CGMH) and Kaohsiung Medical University Chung-Ho Memorial Hospital (KMUH).

Feature	Kaohsiung Chang Gung Memorial Hospital KD Training Set		Kaohsiung Medical University Chung-Ho Memorial Hospital KD Validation Set	
	Non- Discretized (Original Data)	Feature- Discretized (Feature Augmentation)	Non- Discretized (Original Data)	Feature- Discretized (Feature Augmentation)
WBC	11.10	0.30	1.36	0.51
RBC	−0.31	0.50	0.81	1.29
Hemoglobin	0.96	0.06	−0.64	0.04
Hematocrit	0.88	0.39	−0.51	0.34
MCH	0.30	0.74	−1.81	0.68
MCHC	−0.68	−0.25	−0.54	−0.16
RDW	2.11	1.16	3.84	2.23
Platelets	1.09	−0.23	1.07	0.11
Segment	−0.07	−0.66	−0.39	−1.09
Band	6.25	1.32	3.06	1.03
Lymphocyte	0.28	0.87	0.67	1.68
Monocyte	1.74	−0.07	1.08	−0.17
Eosinophil	4.15	0.25	10.19	1.06
Basophil	6.53	1.38	4.44	1.47
AST	114.53	0.84	18.85	1.77
ALT	78.73	3.67	10.20	4.10
CRP	3.76	1.62	2.92	1.32
UWBC	5.35	−1.26	5.29	3.30

**Table 6:** Performance comparison across CTGAN-augmented models with and without feature discretization on two Kawasaki Disease datasets from Chang Gung Memorial Hospital (CGMH) and Kaohsiung Medical University Hospital (KMUH).

Metric	Kaohsiung Chang Gung Memorial Hospital KD Test Set				Kaohsiung Medical University Chung-Ho Memorial Hospital KD Validation Set			
	Non-Discretized (Original Data)		Feature-Discretized (Feature Augmentation)		Non-Discretized (Original Data)		Feature-Discretized (Feature Augmentation)	
	XGBoost	Stacking Model	Stacking Model	FADEL Model	XGBoost	Stacking Model	Stacking Model	FADEL Model
Recall/Sensitivity (%)	80.8	96.1	97.4	95.6	80.6	85.5	91.9	90.3
F1 Score (%)	69.7	47.1	40.2	47.8	40.2	32.8	19.5	27.5
G-mean (%)	89.5	96.4	96.4	96.2	85.7	85.9	79.8	85.6
Specificity (%)	99.2	96.7	95.5	96.8	91.0	86.3	69.3	80.9

**Table 7:** Kawasaki Disease prediction: performance comparison of the FADEL framework with existing methods.

Authors	Method	Study Population	KD Datasets *N* (Positive, Negative)	Sensitivity	Specificity	G-Mean
Maki et al., 2018 [48]	Logistic Regression	Japan	129 (37, 92)	86%	86%	86%
Lam et al., 2022 [49]	Feedforward Neural Networks	USA	1517 (775, 742)	92%	95%	93%
Xu et al., 2022 [50]	Convolutional Neural Network	-	2035 (1023, 1012)	80%	85%	82%
Li et al., 2023 [51]	Logistic Regression	China	608 (299, 309)	86%	84%	84%
Portman et al., 2023 [52]	Least-Angle Regression	USA	150 (50, 100)	92%	86%	88%
Fabi et al., 2023 [53]	Clinical Score and Clinic-Lab Score	Italy	90 (34, 56)	98%	83%	90%
**FADEL**	**Ensemble Learning with** **Feature Augmentation**	**Taiwan**	**79,400 (1230, 78,170)**	**91%**	**98%**	**95%**

The lack of publicly available implementation models in the referenced studies [48,49,50,51,52,53] rendered external validation on the KMUH dataset infeasible. Accordingly, a method-level comparison was conducted based on their reported results, with G-mean adopted as the primary evaluation metric to ensure a fair and consistent assessment on highly imbalanced datasets.

## Data Availability

The real medical datasets are not available due to privacy restrictions.

## References

[1] He H., Garcia E.A. (2009). Learning from Imbalanced Data. IEEE Trans. Knowl. Data Eng..

[2] Galar M., Fernandez A., Barrenechea E., Bustince H., Herrera F. (2012). A Review on Ensembles for the Class Imbalance Problem: Bagging-, Boosting-, and Hybrid-Based Approaches. IEEE Trans. Syst. Man Cybern. C Appl. Rev..

[3] Mathew R.M., Gunasundari R. A Review on Handling Multiclass Imbalanced Data Classification in Education Domain. Proceedings of the 2021 International Conference on Advance Computing and Innovative Technologies in Engineering (ICACITE).

[4] Su Q., Hamed H.N.A., Isa M.A., Hao X., Dai X. (2024). A GAN-Based Data Augmentation Method for Imbalanced Multi-Class Skin Lesion Classification. IEEE Access.

[5] Edward J., Rosli M.M., Seman A. (2023). A New Multi-Class Rebalancing Framework for Imbalance Medical Data. IEEE Access.

[6] Aftabi S.Z., Ahmadi A., Farzi S. (2023). Fraud Detection in Financial Statements Using Data Mining and GAN Models. Expert Syst. Appl..

[7] Li X., Wu X., Wang T., Xie Y., Chu F. (2025). Fault Diagnosis Method for Imbalanced Data Based on Adaptive Diffusion Models and Generative Adversarial Networks. Eng. Appl. Artif. Intell..

[8] Basha S.J., Madala S.R., Vivek K., Kumar E.S., Ammannamma T. A Review on Imbalanced Data Classification Techniques. Proceedings of the 2022 International Conference on Advanced Computing Technologies and Applications (ICACTA).

[9] Salmi M., Atif D., Oliva D., Abraham A., Ventura S. (2024). Handling Imbalanced Medical Datasets: Review of a Decade of Research. Artif. Intell. Rev..

[10] Chawla N.V., Bowyer K.W., Hall L.O., Kegelmeyer W.P. (2002). SMOTE: Synthetic Minority Over-Sampling Technique. J. Artif. Intell. Res..

[11] He H., Bai Y., Garcia E.A., Li S. ADASYN: Adaptive Synthetic Sampling Approach for Imbalanced Learning. Proceedings of the 2008 IEEE International Joint Conference on Neural Networks (IEEE World Congress on Computational Intelligence).

[12] Goodfellow I.J., Pouget-Abadie J., Mirza M., Xu B., Warde-Farley D., Ozair S., Courville A., Bengio Y. (2014). Generative Adversarial Nets. Advances in Neural Information Processing Systems.

[13] Xu L., Skoularidou M., Cuesta-Infante A., Veeramachaneni K., Wallach H., Larochelle H., Beygelzimer A., d’Alché-Buc F., Fox E., Garnett R. (2019). Modeling Tabular Data Using Conditional GAN. Advances in Neural Information Processing Systems.

[14] Chen W., Yang K., Yu Z., Shi Y., Chen C.L.P. (2024). A Survey on Imbalanced Learning: Latest Research, Applications and Future Directions. Artif. Intell. Rev..

[15] Johnson J.M., Khoshgoftaar T.M. (2019). Survey on Deep Learning with Class Imbalance. J. Big Data.

[16] Pavlyshenko B. Using Stacking Approaches for Machine Learning Models. Proceedings of the 2018 IEEE Second International Conference on Data Stream Mining & Processing (DSMP).

[17] Lu M., Hou Q., Qin S., Zhou L., Hua D., Wang X., Cheng L. (2023). A Stacking Ensemble Model of Various Machine Learning Models for Daily Runoff Forecasting. Water.

[18] Ghasemieh A., Lloyed A., Bahrami P., Vajar P., Kashef R. (2023). A Novel Machine Learning Model with Stacking Ensemble Learner for Predicting Emergency Readmission of Heart-Disease Patients. Decis. Anal. J..

[19] Loch-Olszewska H., Szwabiński J. (2020). Impact of Feature Choice on Machine Learning Classification of Fractional Anomalous Diffusion. Entropy.

[20] van der Walt C.M., Barnard E. (2007). Data Characteristics That Determine Classifier Performance. SAIEE Afr. Res. J..

[21] Chen T., Guestrin C. (2016). XGBoost: A Scalable Tree Boosting System. Proceedings of the 22nd ACM SIGKDD International Conference on Knowledge Discovery and Data Mining (KDD’16).

[22] Ke G., Meng Q., Finley T., Wang T., Chen W., Ma W., Ye Q., Liu T.-Y. LightGBM: A Highly Efficient Gradient Boosting Decision Tree. Proceedings of the 31st Conference on Neural Information Processing Systems (NeurIPS 2017).

[23] Prokhorenkova L., Gusev G., Vorobev A., Dorogush A.V., Gulin A. CatBoost: Unbiased Boosting with Categorical Features. Proceedings of the 32nd International Conference on Neural Information Processing Systems (NeurIPS 2018).

[24] Freund Y., Schapire R.E. (1997). A Decision-Theoretic Generalization of On-Line Learning and an Application to Boosting. J. Comput. Syst. Sci..

[25] Mujahid M., Kına E., Rustam F., Villar M.G., Alvarado E.S., Diez I.D.L.T., Ashraf I. (2024). Data Oversampling and Imbalanced Datasets: An Investigation of Performance for Machine Learning and Feature Engineering. J. Big Data.

[26] Wang S., Dai Y., Shen J., Xuan J. (2021). Research on Expansion and Classification of Imbalanced Data Based on SMOTE Algorithm. Sci. Rep..

[27] Alkhawaldeh I.M., Albalkhi I., Naswhan A.J. (2023). Challenges and Limitations of Synthetic Minority Oversampling Techniques in Machine Learning. World J. Methodol..

[28] Mukherjee M., Khushi M. (2021). SMOTE-ENC: A Novel SMOTE-Based Method to Generate Synthetic Data for Nominal and Continuous Features. Appl. Syst. Innov..

[29] Zhu T., Liu X., Zhu E. (2023). Oversampling with Reliably Expanding Minority Class Regions for Imbalanced Data Learning. IEEE Trans. Knowl. Data Eng..

[30] Majeed A., Hwang S.O. (2023). CTGAN-MOS: Conditional Generative Adversarial Network Based Minority-Class-Augmented Oversampling Scheme for Imbalanced Problems. IEEE Access.

[31] Yan Y., Zhu Y., Liu R., Zhang Y., Zhang Y., Zhang L. (2023). Spatial Distribution-Based Imbalanced Undersampling. IEEE Trans. Knowl. Data Eng..

[32] Wang Z., Cao C., Zhu Y. (2020). Entropy and Confidence-Based Undersampling Boosting Random Forests for Imbalanced Problems. IEEE Trans. Neural Netw. Learn. Syst..

[33] Arefeen M.A., Nimi S.T., Rahman M.S. (2022). Neural Network-Based Undersampling Techniques. IEEE Trans. Syst. Man Cybern. Syst..

[34] Liu H., Hussain F., Tan C.L., Dash M. (2002). Discretization: An Enabling Technique. Data Min. Knowl. Discov..

[35] Rajbahadur G.K., Wang S., Kamei Y., Hassan A.E. (2021). Impact of Discretization Noise of the Dependent Variable on Machine Learning Classifiers in Software Engineering. IEEE Trans. Softw. Eng..

[36] García S., Luengo J., Sáez J.A., López V., Herrera F. (2013). A Survey of Discretization Techniques: Taxonomy and Empirical Analysis in Supervised Learning. IEEE Trans. Knowl. Data Eng..

[37] Thaiphan R., Phetkaew T. Comparative Analysis of Discretization Algorithms on Decision Tree. Proceedings of the 2018 IEEE/ACIS 17th International Conference on Computer and Information Science (ICIS).

[38] Xie Z.-H., Wu W.-Z., Wang L.-X. Optimal Scale Selection in Multi-Scale Interval-Set Decision Tables. Proceedings of the 2023 International Conference on Machine Learning and Cybernetics (ICMLC).

[39] Liu X., Wang H. (2005). A Discretization Algorithm Based on a Heterogeneity Criterion. IEEE Trans. Knowl. Data Eng..

[40] Alazaidah R. A Comparative Analysis of Discretization Techniques in Machine Learning. Proceedings of the 2023 24th International Arab Conference on Information Technology (ACIT).

[41] Blessie E.C., Karthikeyan E. RELIEF-DISC: An Extended RELIEF Algorithm Using Discretization Approach for Continuous Features. Proceedings of the 2011 Second International Conference on Emerging Applications of Information Technology (EAIT).

[42] Zhang Y., Deng L., Wei B. (2024). Imbalanced Data Classification Based on Improved Random-SMOTE and Feature Standard Deviation. Mathematics.

[43] Sagi O., Rokach L. (2018). Ensemble Learning: A Survey. WIREs Data Min. Knowl. Discov..

[44] Mienye I.D., Sun Y. (2022). A Survey of Ensemble Learning: Concepts, Algorithms, Applications, and Prospects. IEEE Access.

[45] Altalhan M., Algarni A., Turki-Hadj Alouane M. (2025). Imbalanced Data Problem in Machine Learning: A Review. IEEE Access.

[46] Zhu T., Hu X., Liu X., Zhu E., Zhu X., Xu H. (2025). Dynamic Ensemble Framework for Imbalanced Data Classification. IEEE Trans. Knowl. Data Eng..

[47] Iosifidis V., Papadopoulos S., Rosenhahn B., Ntoutsi E. (2023). AdaCC: Cumulative Cost-Sensitive Boosting for Imbalanced Classification. Knowl. Inf. Syst..

[48] Maki H., Maki Y., Shimamura Y., Fukaya N., Ozawa Y., Shibamoto Y. (2019). Differentiation of Kawasaki disease from other causes of fever and cervical lymphadenopathy: A diagnostic scoring system using contrast-enhanced CT. Am. J. Roentgenol..

[49] Lam J.Y., Chen Z., Wang X., Liu Y., Sun L., Gong F. (2022). A machine-learning algorithm for diagnosis of multisystem inflammatory syndrome in children and Kawasaki disease in the USA: A retrospective model development and validation study. Lancet Digit. Health.

[50] Xu E., Nemati S., Tremoulet A.H. (2022). A deep convolutional neural network for Kawasaki disease diagnosis. Sci. Rep..

[51] Li C., Liu Y., Liu D., Wang W., Wang J., Hu Y. (2023). A machine learning model for distinguishing Kawasaki disease from sepsis. Sci. Rep..

[52] Portman M.A., Magaret C.A., Barnes G., Peters C., Rao A., Rhyne R. (2023). An artificial intelligence derived blood test to diagnose Kawasaki disease. Hosp. Pediatr..

[53] Fabi M., Dondi A., Andreozzi L., Frazzoni L., Biserni G.B., Ghiazza F., Dajti E., Zagari R.M., Lanari M. (2023). Kawasaki disease, multisystem inflammatory syndrome in children, and adenoviral infection: A scoring system to guide differential diagnosis. Eur. J. Pediatr..

